# Managers’ sick-leave recommendations - a video vignette study on common mental disorders

**DOI:** 10.1093/eurpub/ckac130.125

**Published:** 2022-10-25

**Authors:** G Hensing, J Hultquist, M Bertilsson

**Affiliations:** 1 School of Public Health and Community Medicine, University of Gothenburg, Göteborg, Sweden; 2 Department of Health and Rehabilitation, University of Gothenburg, Göteborg, Sweden

## Abstract

**Background:**

Sick leave due to common mental disorders (CMD) in Sweden is higher among women than men. Since the Swedish labor market is highly sex segregated a contributing factor might be managers’ attitudes towards CMD and sick leave. This video vignette study tests three hypotheses on managerś sex and recommendation for sick leave. The hypotheses are: (1) there is an association between negative attitudes towards CMDs and recommending sick leave, and (2) there is an association between educational level and recommending sick leave, and (3) there is an association between workplace factors and managerś recommendation of sick leave.

**Methods:**

The study sample consisted of 2703 Swedish managers, female (34%) and male (66%). The online survey included a randomized female and male video vignette played by actors and specifically designed for the study. Associations were investigated by means of logistic regression. The covariates were attitudes towards depression, educational level, labor sector, size of company, proportion of women/men at the workplace, and sex of the person in the video vignette.

**Results:**

The bi-variate crude analysis showed an OR of 1.28 (95% C.I. 1.08-1.51) for female vs. male managers’ recommendation of employee sick leave to the video vignette. Negative attitudes towards CMD did not add to the model, whereas educational level did, OR 1.34 (95% C.I. 1.13-1.59). The final, fully adjusted model showed an OR of 1.39 (95% C.I. 1.16-1.66) for female vs. male managers’ recommendation of employee sick leave.

**Conclusions:**

The likelihood of a manager recommending sick leave after watching a CMD-labelled video vignette was slightly higher for female managers compared to male, and it remained in the final adjusted model. The results resonate with the registered sick leave and the sex segregation among managers and industries in the Swedish labor market.

**Key messages:**

- Female managers were slightly more likely to recommend sick leave to a video vignette case compared to male managers.
- Negative attitudes towards CMD were not associated with recommending sick leave.

